# Modeling Ebola Virus Transmission Using Ferrets

**DOI:** 10.1128/mSphere.00309-18

**Published:** 2018-10-31

**Authors:** Marc-Antoine de La Vega, Geoff Soule, Kaylie N. Tran, Kevin Tierney, Shihua He, Gary Wong, Xiangguo Qiu, Gary P. Kobinger

**Affiliations:** a Département de microbiologie-infectiologie et d’immunologie, Université Laval, Quebec City, Quebec, Canada; b Special Pathogens Program, National Microbiology Laboratory, Public Health Agency of Canada, Winnipeg, Manitoba, Canada; c Department of Medical Microbiology, University of Manitoba, Winnipeg, Manitoba, Canada; d Guangdong Key Laboratory for Diagnosis and Treatment of Emerging Infectious Diseases, Shenzhen Key Laboratory of Pathogen and Immunity, Shenzhen Third People’s Hospital, Shenzhen, China; e Department of Pathology and Laboratory Medicine, University of Pennsylvania School of Medicine, Philadelphia, Pennsylvania, USA; Boston University School of Medicine

**Keywords:** animal models, Ebola virus, ferret, filovirus, transmission

## Abstract

Ebola virus (EBOV) has been responsible for sporadic outbreaks in Central Africa since 1976 and has the potential of causing social disruption and public panic as illustrated by the 2013–2016 epidemic in West Africa. Transmission of EBOV has been described to occur via contact with infected bodily fluids, supported by data indicating that infectious EBOV could be cultured from blood, semen, saliva, urine, and breast milk. Parameters influencing transmission of EBOV are, however, largely undefined in part due to the lack of an established animal model to study mechanisms of pathogen spread. Here, we investigated EBOV transmissibility in male and female ferrets. After intranasal challenge, an infected animal was placed in direct contact with a naive ferret and in contact with another naive ferret (separated from the infected animal by a metal mesh) that served as the indirect-contact animal. All challenged animals, male direct contacts, and one male indirect contact developed disease and died. The remaining animals were not viremic and remained asymptomatic but developed EBOV-glycoprotein IgM and/or IgG specific antibodies—indicative of virus transmission. EBOV transmission via indirect contact was frequently observed in this model but resulted in less-severe disease compared to direct contact. Interestingly, these observations are consistent with the detection of specific antibodies in humans living in areas of EBOV endemicity.

**IMPORTANCE** Our knowledge regarding transmission of EBOV between individuals is vague and is mostly limited to spreading via direct contact with infectious bodily fluids. Studying transmission parameters such as dose and route of infection is nearly impossible in naturally acquired cases—hence the requirement for a laboratory animal model. Here, we show as a proof of concept that ferrets can be used to study EBOV transmission. We also show that transmission in the absence of direct contact is frequent, as all animals with indirect contact with the infected ferrets had detectable antibodies to the virus, and one succumbed to infection. Our report provides a new small-animal model for studying EBOV transmission that does not require adaptation of the virus, providing insight into virus transmission among humans during epidemics.

## Abstract (precis)

Our knowledge regarding transmission of EBOV between individuals is vague and is mostly limited to spreading via direct contact with infectious bodily fluids. Studying transmission parameters such as dose and route of infection is nearly impossible in naturally acquired cases—hence the requirement for a laboratory animal model. Here, we show as a proof of concept that ferrets can be used to study EBOV transmission. We also show that transmission in the absence of direct contact is frequent, as all animals with indirect contact with the infected ferrets had detectable antibodies to the virus, and one succumbed to infection. Our report provides a new small-animal model for studying EBOV transmission that does not require adaptation of the virus, providing insight into virus transmission among humans during epidemics.

## INTRODUCTION

In March 2014, an Ebola virus (EBOV) disease (EVD) outbreak was declared in Guéckédou, Guinea, and rapidly spread to Sierra Leone and Liberia (1). As of 27 March 2016, 28,646 cases of EVD were reported, with 11,323 deaths in 10 countries, making it the biggest and most widespread EVD epidemic to date. According to the World Health Organization, many factors played substantial roles in virus propagation, one of which was that West African EBOV isolates may possess clinical and epidemiological features that differ from those of previous strains (2). Extensive pathogenicity studies have been conducted with Central African isolates of EBOV, while West African EBOV isolates (EBOV-Makona) were also investigated in nonhuman primates (NHPs). A previous pathogenicity study found that EBOV-Makona, isolate C07, caused delayed disease progression in cynomolgus macaques compared to the reference EBOV-Mayinga isolate (collected from the 1976 outbreak in Central Africa), suggesting decreased virulence of this isolate (3). In contrast, another study in rhesus macaques showed that EBOV-Makona, isolate C05, could replicate to higher titers than C07 and the reference EBOV-Kikwit strain (isolated from the 1995 outbreak in Central Africa). Monkeys infected by EBOV-Makona-C05 or EBOV-Makona-C07 also experienced more-severe organ damage and shed on average 2-fold to 16-fold more virus via the oral, nasal, and rectal routes, suggesting that animals infected with these West African isolates were more infectious (4).

It is well accepted that human-to-human transmission of EBOV in outbreaks occurs through direct contact with infected bodily fluids (5, 6); however, there have been few studies on EBOV transmission in animal models. A better understanding of all the potential modes of EBOV transmission is important for protecting the general population and clinical personnel against infection. Transmission of EBOV-Mayinga from infected to naive rhesus macaques in the absence of direct contact has been previously described (7), but similar conditions involving EBOV-Kikwit and cynomolgus macaques did not result in transmission (8). Pig-to-pig (9) and pig-to-NHP (10) transmissions have been described for EBOV-Kikwit, and a guinea pig model of EBOV transmission was established using guinea pig-adapted EBOV-Mayinga, the first rodent model for transmission studies (11).

## RESULTS

**FIG 1:**
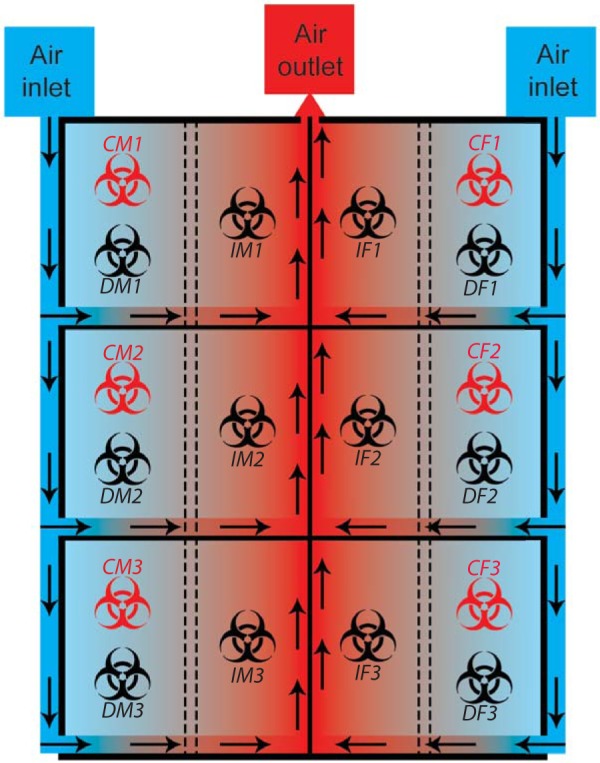
Experimental setting of the transmission study. Challenged animals were placed on the outer half of the divided units along with a nonchallenged, direct-contact animal. Indirect-contact animals were placed in the inner half of the units. Directional airflow is illustrated by arrows, where clean air was introduced in the caging system from the outer half of the unit, passed through each cage, and evacuated through the middle section of the system. Challenged animals are represented by red biohazard symbols, while black biohazard symbols represent nonchallenged animals. Male ferrets were located on the left, and female ferrets were on the right.

**FIG 2:**
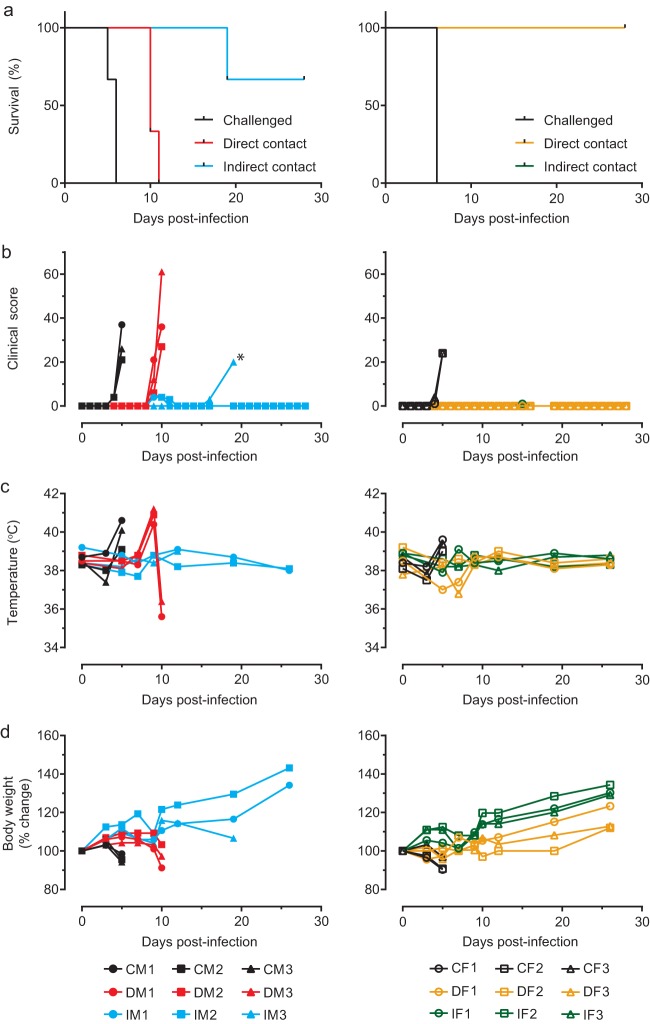
Survival and clinical parameters of challenged and contact ferrets. Clinical parameters of male and female ferrets challenged or not with EBOV-Makona-C05 are presented. (a) Survival. (b) Temperature. (c) Clinical score. (d) Body weight percent change. Key: C, challenged animal; D, direct-contact animal; I, indirect-contact animal; M, male (left column); F, female (right column); *, animal was found dead and could not be scored.

Recent pathogenesis studies in domestic ferrets with EBOV, as well as other filoviruses, showed that ill animals shed high amounts of live virus (12–14). Here, we demonstrated the potential for using domesticated ferrets to study direct and indirect EBOV transmission with EBOV-Makona, isolate C05. In this study, animals were caged such that transmission via direct and indirect contact could be investigated simultaneously. The direct-contact animal was placed on one side of the cage along with the challenged animal, while the indirect-contact animal was placed downstream (of the airflow) of the first two animals, separated from them by a mesh preventing physical contact between the animals on the two sides of the mesh (Fig. 1). Challenged male (CM1 to CM3) and female (CF1 to CF3) ferrets were infected intranasally (i.n.) with 1,000× the 50% tissue culture infective dose (TCID_50_) of EBOV-Makona to mimic mucosal exposure. All challenged males and females succumbed to infection, with mean times to death of 5.7 (standard deviation [SD], 0.6 days; range, 5.0 to 6.0 days) and 6.0 days, respectively. This difference was not statistically significant (two-tailed *t* test with Welch’s correction; *t* = 1, df = 2). All direct-contact males (DM1 to DM3) showed disease symptoms and died with a mean time to death of 10.3 days (SD, 0.6; range, 10 to 11 days), whereas all direct-contact females (DF1 to DF3) survived. One indirect-contact male animal (IM3) was found dead on day 19, whereas the other indirect-contact animals (IM1 and IM2) survived. All females in the indirect-contact group (IF1 to IF3) survived (Fig. 2a). The surviving animals did not exhibit any EVD-associated symptoms at any point throughout the experiment, unlike the animals that succumbed (Fig. 2b). The animals in the latter category typically had a spike in body temperature the day before death (Fig. 2c) and experienced mild to moderate weight loss (Fig. 2d). While all challenged animals died, male ferrets exhibited more-severe EVD symptoms, such as a petechial rash and fever (see Table S1 to S3 in the supplemental material). Of note, rectal temperatures were taken solely on sampling days in order to minimize stress to the animals; therefore, days with fever could have been missed. Changes in blood biochemistry and complete blood counts after infection in male and female animals were comparable (see Fig. S1 and S2 in the supplemental material).

10.1128/mSphere.00309-18.1 FIG S1 Complete blood counts and biochemical parameters of challenged and contact male ferrets. Complete blood counts and biological parameters of male ferrets challenged or not with EBOV-Makona-C05 are presented. a, white blood cell count; b, lymphocyte count; c, lymphocyte percentage; d, platelet count; e, neutrophil count; f, neutrophil percentage; g, alkaline phosphatase; h, alanine aminotransferase; i, blood urea nitrogen; j, creatinine; k, glucose. Key: C, challenged animal; D, direct-contact animal; I, indirect-contact animal; M, male. Download FIG S1, PDF file, 0.6 MB. © Crown copyright 2018. 2018 Crown This content is distributed under the terms of the Creative Commons Attribution 4.0 International license.

10.1128/mSphere.00309-18.2 FIG S2 Complete blood counts and biochemical parameters of challenged and contact female ferrets. Complete blood counts and biological parameters of female ferrets challenged or not with EBOV-Makona-C05 are presented. a, white blood cell count; b, lymphocyte count; c, lymphocyte percentage; d, platelet count; e, neutrophil count; f, neutrophil percentage; g, alkaline phosphatase; h, alanine aminotransferase; i, blood urea nitrogen; j, creatinine; k, glucose. Key: C, challenged animal; D, direct-contact animal; I, indirect-contact animal; F, female. Download FIG S2, PDF file, 0.6 MB. © Crown copyright 2018. 2018 Crown This content is distributed under the terms of the Creative Commons Attribution 4.0 International license.

10.1128/mSphere.00309-18.3 TABLE S1 Clinical parameters of male ferrets. Hypothermia was defined as exposure to temperatures below 35°C. Fever was defined as body temperature 1.0°C higher than baseline. Mild rash was defined as focal areas of petechiae covering <10% of the skin, moderate rash as areas of petechiae covering 10 to 40% of the skin, and severe rash as areas of petechiae and/or ecchymosis covering >40% of the skin. Leukocytopenia and thrombocytopenia were defined as a >30% decrease in numbers of white blood cells and platelets, respectively. Leukocytosis and thrombocytosis were diagnosed with measurements of a 2-fold or greater increase in numbers of white blood cells and platelets over baseline and white blood cell count of >11 × 10^3^. ↑, 2-fold to 3-fold increase; ↑↑, 4-fold to 5-fold increase; ↑↑↑, greater than 5-fold increase; ↓, 2-fold to 3-fold decrease; ↓↓, 4-fold to 5-fold decrease; ↓↓↓, greater than 5-fold decrease. AMY, amylase; ALP, alkaline phosphatase; ALT, alanine aminotransferase; TBIL, total bilirubin; BUN, blood urea nitrogen; CRE, creatinine; K+, potassium; GLOB, globulin. Download Table S1, PDF file, 0.1 MB. © Crown copyright 2018. 2018 Crown This content is distributed under the terms of the Creative Commons Attribution 4.0 International license.

All challenged animals succumbed to infection within a time period consistent with past reports of EVD in ferrets, which has been described to be 5 to 6 days (12–14). Among the direct-contact and indirect-contact animals, DM1, DM2, and DM3 succumbed to infection 4 to 6 days after CM1, CM2, and CM3 died, suggesting that transmission likely occurred when the challenged ferrets had advanced or terminal EVD. This is consistent with current knowledge, as viral loads are usually highest at the time of death, suggesting that severely ill animals are the most infectious (3, 12, 15, 16). Ferret IM3 died 9 days after DM3 succumbed to infection, suggesting that IM3 might have acquired the infection during DM3’s late stage of disease but might also have been infected by infectious fomites from CM3 or DM3. While direct physical contact was not possible between animals separated by the mesh, small particles might have passed through the holes to infect indirect-contact animals. This is consistent with findings from a previous study using a similar experimental setup showing that fomites, such as soiled bedding, were important for promoting EBOV transmission in guinea pigs (11).

**FIG 3:**
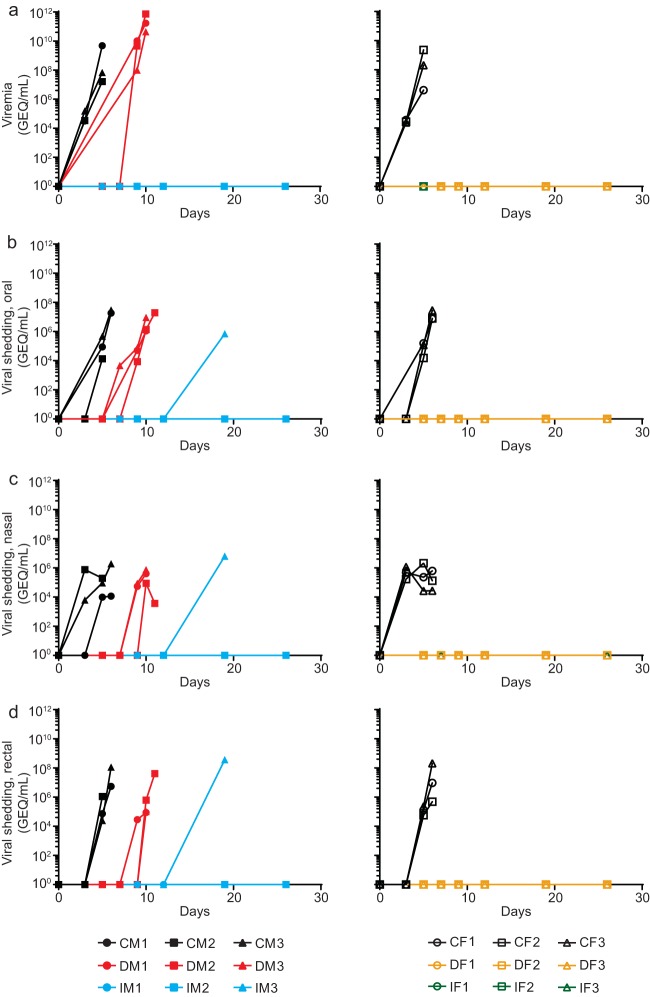
Viral loads and shedding from all animals. Viral loads are measured by RT-qPCR in the blood of male and female ferrets (a), oral swabs (b), nasal washes (c), and rectal swabs (d). Key: C, challenged animal; D, direct-contact animal; I, indirect-contact animal; M, male (left column); F, female (right column). Note that a blood sample was not available from animal IM3 on day 19 as it was found dead.

As infection progressed, animals were evaluated for viremia and virus shedding through the oral, nasal, and rectal mucosae. The average peak viremia levels were 1.79E9 genome equivalent copies (GEQ)/ml and 8.4E8 GEQ/ml for CM1 to CM3 and CF1 to CF3, respectively. The difference was not found to be statistically different [one-way analysis of variance (ANOVA); F(3,6) = 0.4677]. DM1 to DM3 had a mean peak viremia level of 3.2E11 GEQ/ml, while IM3 did not have an available sample since it was found dead (Fig. 3a). However, EBOV titers from postmortem oral, nasal, and rectal swabs for IM3 were 7.1E5, 6.4E6, and 3.7E8 GEQ/ml, respectively. The average peak levels of oral, nasal, and rectal shedding for CM1 to CM3 were 1.6E7 GEQ/ml, 9.0E5 GEQ/ml, and 4.0E7 GEQ/ml, respectively. Similarly, CF1 to CF3 exhibited average peak levels of oral, nasal, and rectal shedding of 1.4E7 GEQ/ml, 1.3E6 GEQ/ml, and 7.2E7 GEQ/ml, respectively (Fig. 3b to d). Environmental swabs on the front and back of the metal mesh and the air exhaust on the side of the indirect-contact animals were negative for EBOV by reverse transcription real-time quantitative PCR (RT-qPCR), with the exception of the air exhaust by IM2 on day 7, which was positive for EBOV at 2.3E3 GEQ/ml. These results indicate that low levels of EBOV can be detected in the surroundings of indirect-contact animals, supporting the hypothesis that IM3 was most likely infected by fomites.

**FIG 4:**
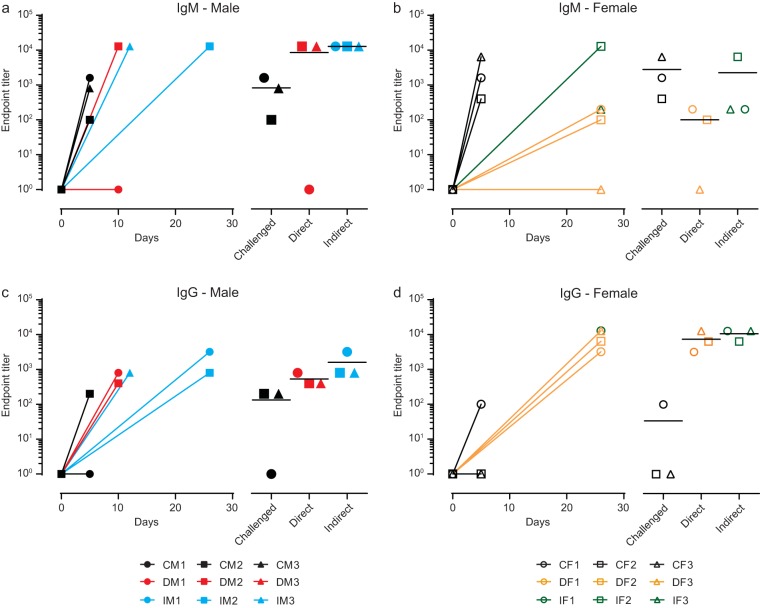
Humoral response of challenged and contact ferrets. Data represent endpoint titers of IgM (a and b) and IgG (c and d) antibodies against the glycoprotein of EBOV in the serum of challenged and contact ferrets at euthanasia. C, challenged animal; D, direct-contact animal; I, indirect-contact animal; M, male (left column); F, female (right column).

All animals were assessed for the presence of EBOV glycoprotein-specific immunoglobulin M (IgM) and immunoglobulin G (IgG) antibodies at the time of euthanasia. DM1 (day 10) and DF3 (day 26) had IgM antibody levels below the limit of detection of the assay, while all other animals were seropositive by the end of the experiment (Fig. 4a and b). All animals were seropositive for IgG except for CM1 (day 5) and CF2 and CF3 (day 5) (Fig. 4c and d). Therefore, all animals were seropositive for either one or both antibody isotypes at the end of the experiment. These results suggest that EBOV transmission via indirect contact is less efficient than direct contact in inducing EVD. As such, only 1 of 6 indirect-contact ferrets received an exposure dose of live virus sufficiently high to result in EVD (Table S1 and S2) despite the finding that exposure to EBOV via indirect contact was frequent, demonstrated by seroconversion of all ferrets.

10.1128/mSphere.00309-18.4 TABLE S2 Clinical parameters of female ferrets. Hypothermia was defined as exposure to temperatures below 35°C. Fever was defined as body temperature 1.0°C higher than baseline. Mild rash was defined as focal areas of petechiae covering <10% of the skin, moderate rash as areas of petechiae covering 10 to 40% of the skin, and severe rash as areas of petechiae and/or ecchymosis covering >40% of the skin. Leukocytopenia and thrombocytopenia were diagnosed with measurements of a >30% decrease in numbers of white blood cells and platelets, respectively. Leukocytosis and thrombocytosis were diagnosed with measurements of a 2-fold or greater increase in numbers of white blood cells and platelets over baseline, with white blood cell count >11 × 10^3^. ↑, 2-fold to 3-fold increase; ↑↑, 4-fold to 5-fold increase; ↑↑↑, greater than 5-fold increase; ↓, 2-fold to 3-fold decrease; ↓↓, 4-fold to 5-fold decrease; ↓↓↓, greater than 5-fold decrease. AMY, amylase; ALP, alkaline phosphatase; ALT, alanine aminotransferase; TBIL, total bilirubin; BUN, blood urea nitrogen; CRE, creatinine; K+, potassium; GLOB, globulin. Download Table S2, PDF file, 0.1 MB. © Crown copyright 2018. 2018 Crown This content is distributed under the terms of the Creative Commons Attribution 4.0 International license.

## DISCUSSION

Multiple serosurveys conducted in countries where outbreaks have previously occurred have resulted in reports of higher seroprevalence for EBOV-specific antibodies in human populations residing in forested areas (17–19). A meta-analysis of 51 published seroprevalence studies compared the levels of specific antibodies between asymptomatic participants living with a household or known case contact in an area of EBOV endemicity, living in areas of endemicity but without case contact, or living in areas without known cases of EVD. The analysis showed an estimated seroprevalence of 3.3% in asymptomatic individuals with a household or known case contact. For the other two groups, measured seroprevalence levels were between 0.9% and 17% and between 0% and 24%, respectively, representing a wide range due to the highly heterogeneous nature of the populations, which prevents making an accurate summary estimate (20). Asymptomatic EVD has been described previously in both humans and NHPs (21–24), and a critical factor determining whether disease is symptomatic or asymptomatic may be the exposure dose or the route of infection. Observations in this study are consistent with the presence of specific antibodies to EBOV under conditions of subclinical disease, a phenomenon first reported from serosurveys in humans. Furthermore, recent data in NHPs showed that challenge with a very low dose of EBOV (10 PFU) via the oral route could lead to viral shedding from the nasal route in the absence of clinical pathology (25). However, the experimental group examined in that study was composed of only two animals and the data should thus be interpreted accordingly, but the issue warrants further investigation. In contrast, a recent study of EBOV infection in ferrets has shown that animals challenged with 0.1 or 1 PFU succumbed to the disease at day 6 or 7 postinfection, indicating that a lower challenge dose does not result in an increased time to death. In the same study, an experimental group of 3 animals was also challenged with 0.01 PFU. While 1 animal succumbed to infection on day 7, the other 2 did not develop any symptoms during the course of the experiment (26). This would suggest that IM3, which succumbed to infection 9 days following its cage mate, most likely became infected by fomites after DM3 was removed from the environment. Finally, viral kinetics in guinea pigs have shown that live virus could not be detected in the blood or in oral, nasal, and rectal swabs 1 day postinfection and that virus detection was possible only at day 3 postinfection in nasal and rectal swabs (11).

The lack of overt morbidity and mortality in the female direct-contact animals was surprising, especially considering the outcome seen with their male counterparts. Further investigations performed with larger groups of ferrets, beyond the scope of this current work, are needed to fully understand whether gender plays a role in susceptibility to EBOV in this model. No evidence currently exists in humans to support a biological difference in the levels of male and female susceptibility to EBOV. The fact that women were found to be infected by the virus more often at several outbreaks is more likely linked to an occupational hazard, as, culturally, sub-Saharan Africa women carry a larger share of the responsibility for caring for the ill (27). In the current study, the similarities in shedding between the two sexes and the differences in mortality observed between male and female contact ferrets could have been due to behavioral factors. For example, it is possible that males engaged more in fighting and/or scratching, which would lead to an increased exposure to infected body fluids, such as blood resulting from injuries, hence leading to higher transmission rates.

This is the first reported small-animal model evaluating transmission of a wild-type EBOV-Makona isolate. Understanding the role of infectivity factors such as exposure dose and route of infection involved in EBOV transmission is relevant to public health and outbreak control efforts. These factors also remain a priority in order to update guidelines following an evidence-based process aimed at protecting health care workers, researchers, and the general public from occupational and accidental exposure. It can be seen from this study that both direct contact and indirect contact with severely ill animals can result in EBOV transmission. Moreover, even with indirect exposure being a less likely event, it is an occurrence that can result in fatal EVD under these conditions. The ferret animal model will be valuable in contributing to our understanding of the many parameters that are associated with the transmission of EBOV. They may also help better define conditions that are specific to subclinical disease, which could lead to important findings and influence the development of more-efficient protective, preventive, and curative measures.

## MATERIALS AND METHODS

### Ethics statement.

The experiments described in this study were carried out at the National Microbiology Laboratory (NML) as described in Animal Use Document H-15-029 and were approved by the Animal Care Committee located at the Canadian Science Center for Human and Animal Health, in accordance with the guidelines provided by the Canadian Council on Animal Care. All infectious work was performed inside the biosafety level 4 (BSL4) laboratory at the NML at the Public Health Agency of Canada.

### Animals and challenge.

Eighteen 3-month-old ferrets were purchased from Marshall BioResources. The animals weighed between 600 and 900 g on reception, and there were nine males and nine females. The animals were challenged 8 days after reception with passage 1 of Ebola virus/H. sapiens-wt-GIN/2014/Makona-C05 (GenBank accession no. KT013254) (order *Mononegavirales*, family *Filoviridae*, species *Zaire ebolavirus*). Infection of the animals was performed via the intranasal (i.n.) route, with a targeted dose of 1,000 times the median tissue culture infectious dose (TCID_50_) (backtitered to 464 TCID_50_ per ferret) diluted in a 0.5-ml total volume (0.25 ml per nostril).

### Study design.

Males and females were divided into three aged-matched groups of three same-sex animals, and each group was placed inside a separate unit of the ferret isolator (Allentown Inc., PA, USA). Each unit was divided in half by two metal meshes 2.5 cm apart, which possess 36-mm^2^ holes, preventing direct contact between animals on the two sides of the unit. The airflow in this type of isolator is directed from the outside to the inside and was set at approximately 35 cubic feet per min. The challenged animal (C) and its direct-contact cage mate (D) were placed in the outside half of the cage, while the animal that was in the indirect-contact group (I) was placed in the inside half of the cage. Immediately following challenge, the six infected animals were given time to partially recover from anesthesia, the outer side of each animal’s nose was wiped, and the animals were placed back into the cage with the corresponding contact animal. All animals were monitored for survival, weight loss, temperature, and clinical symptoms (see Table S3 in the supplemental material). Animals were sampled for blood, and oral and rectal swabs as well as nasal washes were collected on days 3, 5, 7, 9, 12, 19, and 26 (end of experiment) to assess levels of viremia and virus shedding and to perform blood biochemistry and complete blood count characterization. Environmental sampling was also conducted over a 10-cm^2^ surface on the front and back of the metal mesh on the side closest to the indirect-contact animal, as well as on the air exhaust located on the side facing the indirect-contact animal.

10.1128/mSphere.00309-18.5 TABLE S3 Ferret humane endpoint scoring chart used for daily assessment of ferrets based on multiple behavioral and clinical criteria. DPI, days postinfection; E/D, eating/drinking. Download Table S3, PDF file, 0.03 MB. © Crown copyright 2018. 2018 Crown This content is distributed under the terms of the Creative Commons Attribution 4.0 International license.

### Serum biochemistry and complete blood count characterization.

Analysis of serum biochemistry was performed using a VetScan VS2 analyzer along with the Comprehensive Diagnostic Profile disk (Abaxis Veterinary Diagnostics), per manufacturer instructions. Analysis of complete blood counts was performed using a VetScan HM5 hematology system (Abaxis Veterinary Diagnostics), per manufacturer instructions.

### EBOV quantification by RT-qPCR.

Oral, rectal, and environmental swabs, as well as nasal washes, were collected in 1 ml of Dulbecco’s modified Eagle’s medium (DMEM), and RNA was extracted from 140 µl of this solution within hours of collection. For titers measured by RT-qPCR, total RNA was extracted from whole blood or DMEM from swab samples with a QIAamp viral RNA minikit (Qiagen). EBOV was detected with a LightCycler 480 RNA Master Hydrolysis Probes (Roche) kit, with the RNA polymerase (nucleotides 16472 to 16538; accession no. AF086833) as the target gene. The reaction conditions were as follows: 63°C for 3 min, 95°C for 30 s, and cycling of 95°C for 15 s and 60°C for 30 s for 45 cycles on an ABI StepOnePlus system. The sequences of the primers used were as follows: for EBOVLF2, CAGCCAGCAATTTCTTCCAT; for EBOVLR2, TTTCGGTTGCTGTTTCTGTG; and for EBOVLP2FAM, 6-carboxyfluorescein (FAM)-ATCATTGGCGTACTGGAGGAGCAG-black hole quencher 1 (BHQ1). GEQ data were determined using the formula *y* = (1,000 × *t* × *n*)/(*b* × *r*), where *t* represents the number of copies in the qPCR reaction (calculated from a standard curve using a plasmid containing the L gene of EBOV), *n* represents the elution volume of the RNA extraction, *b* represents the volume of blood from which the RNA was extracted, and *r* represents the volume of RNA used in the qPCR reaction.

### ELISA.

Enzyme-linked immunosorbent assays (ELISAs) for analysis of Ebola virus glycoprotein (EBOV GP)-specific IgM and IgG were performed using recombinant EbolaGPΔTM (IBT Bioservices catalog no. 0501-025) as the antigen. Briefly, 96-well half-well plates were coated with 30 μl of protein at a concentration of 1 µg/ml, left at 4°C overnight, and then blocked for 1 h at 37°C with phosphate-buffered saline (PBS)–5% skim milk the following morning. Plates were then washed 3 times with 0.1% PBS–Tween, and the serum dilutions were applied to the plates and incubated at 37°C for 1 h followed by washing 3 times with 0.1% PBS–Tween. Goat anti-human IgG-horseradish peroxidase (IgG-HRP) (KPL catalog no. 074-1006) was added for 1 h at 37°C at a 1:1,500 dilution. The plates were read using ABTS [2,2′-azinobis(3-ethylbenzthiazolinesulfonic acid)] peroxidase substrate (Thermo Fisher catalog no. 37615), which was applied at 50 µl per well, and left for 30 min before reading. Each sample was assayed in duplicate. A titer was considered to represent a positive result if the average value was 7.733 standard deviations above background.

### Statistics.

Survival was evaluated using the log rank (Mantel-Cox) test, and time to death was evaluated using the two-tailed, unpaired *t* test with Welch’s correction. Differences between average values of viral loads were evaluated by one-way ANOVA with Tukey’s multiple-comparison test, with single pooled variance. Results were considered significant if the *P* value was <0.05, and all analyses were conducted in GraphPad Prism 7.02.

### Data availability.

The data sets generated during the course of the current study are available from the corresponding author on request.
