# Supplementary figures and images for: Modeling Ebola Virus Transmission Using Ferrets
Source: mSphere. 2018 Oct 31;3(5):e00309-18. doi: 10.1128/mSphere.00309-18 (PMC6211219; doi:10.1128/mSphere.00309-18)

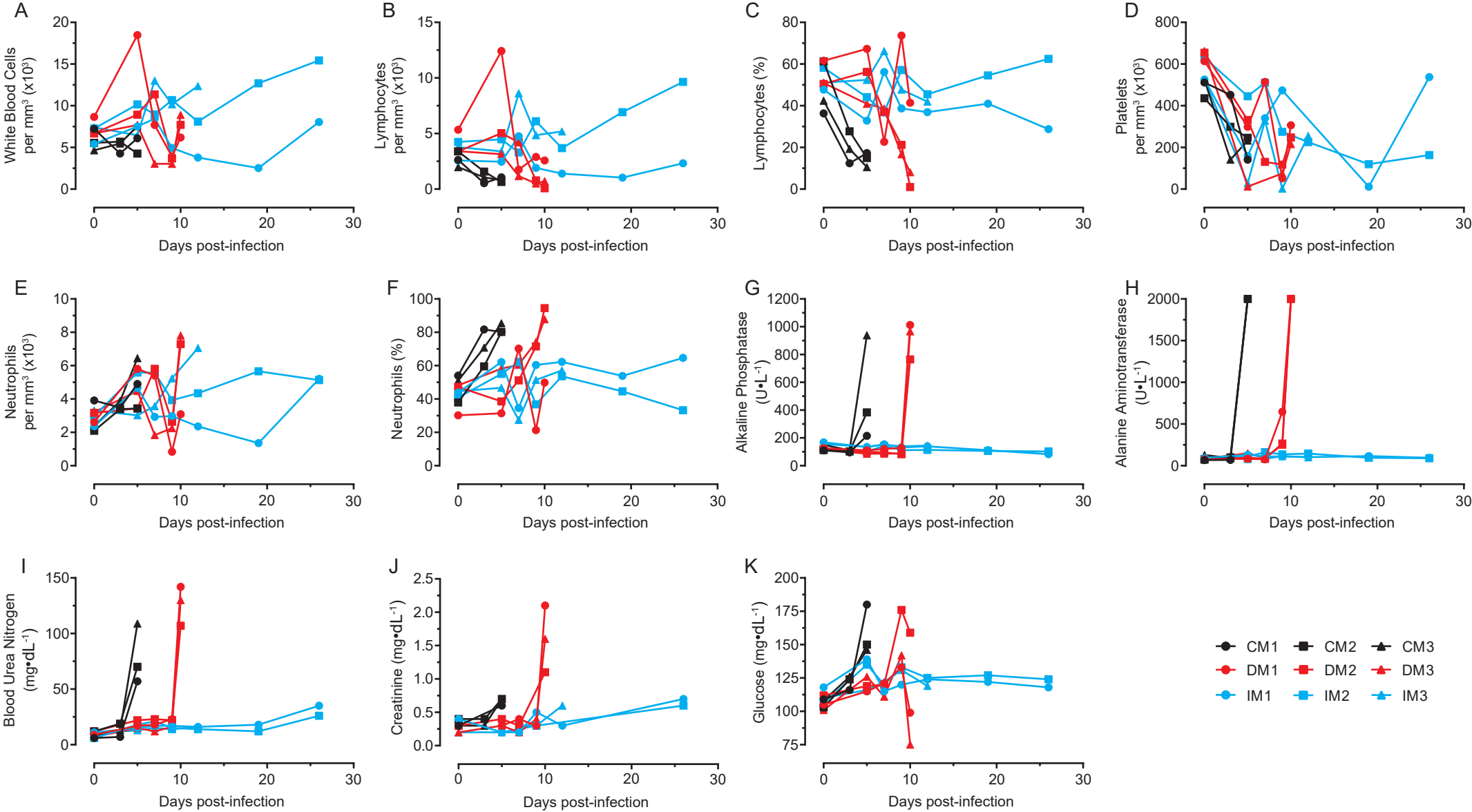

Supplement: FIG S1 [file sph006182689sf1.pdf]

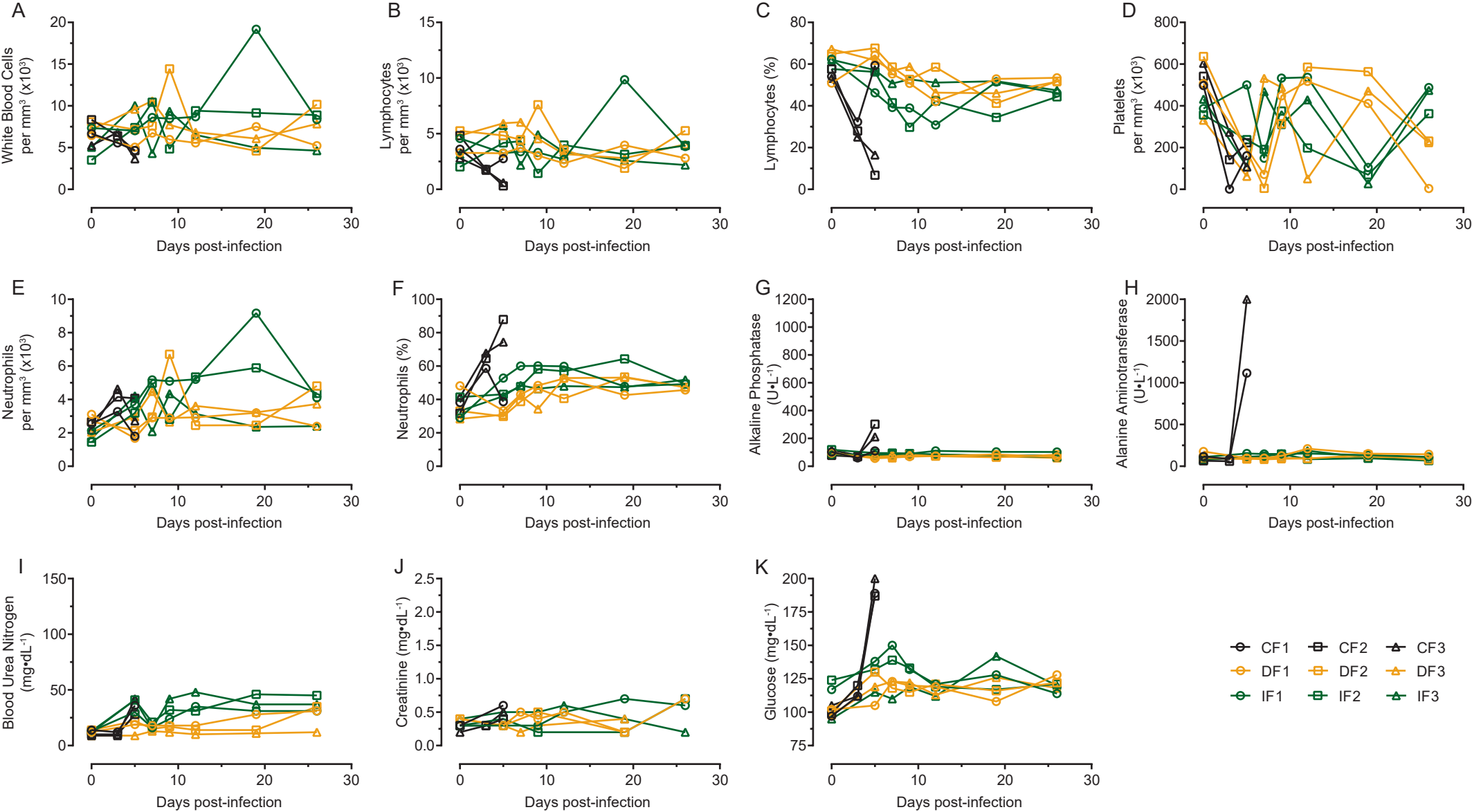

Supplement: FIG S2 [file sph006182689sf2.pdf]
