# Supplementary material for: Modeling Ebola Virus Transmission Using Ferrets
Source: mSphere. 2018 Oct 31;3(5):e00309-18. doi: 10.1128/mSphere.00309-18 (PMC6211219; doi:10.1128/mSphere.00309-18)
Supplement: TABLE S1 [file sph006182689st1.pdf]

Animal ID	Group	Clinical findings					Outcome
		Body temperature	Rash	White blood cells	Platelets	Biochemistry	
**CM1**	Challenged	Fever (5 dpi)	Moderate (5 dpi), Severe (6 dpi)	Leukocytopenia (3, 5 dpi)	Thrombocytopenia (5 dpi)	AMY↑ (3 dpi), ALT↑↑↑, TBIL↑↑↑, BUN↑↑↑, CRE↑ (5 dpi)	Died, 6 dpi
**DM1**	Direct contact	Fever (9 dpi)	Severe (9, 10 dpi)	Leukocytosis (5 dpi), Leukocytopenia (7, 9, 10 dpi)	Thrombocytopenia (5, 9, 10 dpi)	BUN↑, CRE↑ (7 dpi), ALT↑↑↑, TBIL↑, BUN↑ (9 dpi), AMY↑, ALP↑↑↑, ALT↑↑↑, TBIL↑↑↑, BUN↑↑↑, CRE↑↑↑ (10 dpi)	Died, 10 dpi
**IM1**	Indirect Contact			Leukocytopenia (12, 19 dpi)	Thrombocytopenia (5, 7 dpi)	TBIL↑ (19 dpi), ALP↓, BUN↑ (26 dpi)	Survived
**CM2**	Challenged		Severe (5 dpi)	Leukocytopenia (3, 5 dpi)	Thrombocytopenia (3, 5 dpi)	ALP↑, ALT↑↑↑, TBIL↑↑↑, BUN↑↑↑, GLOB↑ (5 dpi)	Died, 5 dpi
**DM2**	Direct contact	Fever (9 dpi)	Severe (10 dpi)	Leukocytopenia (9, 10 dpi)	Thrombocytopenia (5, 7, 9, 10 dpi)	ALT↑, GLOB↑ (9 dpi), ALP↑↑↑, ALT↑↑↑, TBIL↑↑↑, BUN↑↑↑, CRE↑, GLOB↑ (10 dpi)	Died, 11 dpi
**IM2**	Indirect Contact				Thrombocytopenia (9, 12, 19, 26 dpi)	BUN↑ (5, 7, 9, 12, 19 dpi), ALT↑ (7 dpi), BUN↑↑, CRE↑ (26 dpi)	Survived
**CM3**	Challenged	Fever (5 dpi)		Leukocytopenia (3, 5 dpi)	Thrombocytopenia (3, 5 dpi)	ALP↑↑↑, ALT↑↑↑, TBIL↑↑↑, BUN↑↑↑, CRE↑ (5 dpi)	Died, 6 dpi
**DM3**	Direct contact	Fever (9 dpi)	Mild (9 dpi), Severe (10 dpi)	Leukocytopenia (7, 9, 10 dpi)	Thrombocytopenia (5, 9, 10 dpi)	BUN↑ (5 dpi)ALT↑, TBIL↑, BUN↑, CRE↑, GLOB↑ (9 dpi), ALP↑↑↑, ALT↑↑↑, TBIL↑↑↑, BUN↑↑↑, CRE↑↑↑, K^+↑, GLOB↑ (10 dpi)	Died, 10 dpi
**IM3**	Indirect Contact				Thrombocytopenia (7, 12 dpi)	CRE↑↑↑ (12 dpi)	Died, 19 dpi
