# Supplementary material for: Modeling Ebola Virus Transmission Using Ferrets
Source: mSphere. 2018 Oct 31;3(5):e00309-18. doi: 10.1128/mSphere.00309-18 (PMC6211219; doi:10.1128/mSphere.00309-18)
Supplement: TABLE S2 [file sph006182689st2.pdf]

Animal ID	Group	Clinical findings					Outcome
		Body temperature	Rash	White blood cells	Platelets	Biochemistry	
**CF1**	Challenged	Fever (5 dpi)	Severe (5 dpi)	Leukocytopenia (3 dpi)	Thrombocytopenia (3, 5 dpi)	ALT↑↑↑, TBIL↑, BUN↑, CRE↑, GLOB↑ (5 dpi)	Died, 6 dpi
**DF1**	Direct contact				Thrombocytopenia (7, 26 dpi)	TBIL↑ (9, 19, 26 dpi), BUN↑ (19, 26 dpi)	Survived
**IF1**	Indirect Contact			Leukocytopenia (12 dpi)	Thrombocytopenia (7, 19 dpi)	TBIL↑ (12 dpi), BUN↑ (5, 12, 19, 26 dpi), CRE↑ (19, 26 dpi)	Survived
**CF2**	Challenged			Leukocytopenia (3, 5 dpi)	Thrombocytopenia (3, 5 dpi)	ALP↑, ALT↑↑↑, TBIL↑↑↑, BUN↑, GLOB↑ (5 dpi)	Died, 6 dpi
**DF2**	Direct contact			Leukocytopenia (12, 19 dpi)	Thrombocytopenia (7, 9, 26 dpi)	BUN↑ (26 dpi), CRE↓ (19 dpi)	Survived
**IF2**	Indirect Contact				Thrombocytopenia (5, 7, 12, 19 dpi)	TBIL↑ (9, 19 dpi), BUN↑ (5, 9, 12, 19, 26 dpi), CRE↑ (26 dpi)	Survived
**CF3**	Challenged			Leukocytopenia (3, 5 dpi)	Thrombocytopenia (3, 5 dpi)	ALT↑↑↑, TBIL↑↑↑, BUN↑↑, CRE↑, GLOB↑ (5 dpi)	Died, 6 dpi
**DF3**	Direct contact			Leukocytopenia (12, 19 dpi)	Thrombocytopenia (5, 12, 26 dpi)		Survived
**IF3**	Indirect Contact			Leukocytopenia (7, 26 dpi)	Thrombocytopenia (5, 19 dpi)	TBIL↑ (9, 12, 19 dpi), BUN↑ (5, 9, 19, 26 dpi), BUN↑↑ (12 dpi), CRE↓ (26 dpi)	Survived
