# Supplementary material for: Modeling Ebola Virus Transmission Using Ferrets
Source: mSphere. 2018 Oct 31;3(5):e00309-18. doi: 10.1128/mSphere.00309-18 (PMC6211219; doi:10.1128/mSphere.00309-18)
Supplement: TABLE S3 [file sph006182689st3.pdf]

Ferret Humane Endpoint Scoring Chart; **Version 2016-01; Date 2016-11-01**

**Prepared for the week of:** **Animal ID #:**

| Parameter | Degree of parameter | DPI<br>Possible Score | 1<br>Score | 2<br>Score | 3<br>Score | 4<br>Score | 5<br>Score | 6<br>Score | 7<br>Score |
|---|---|---|---|---|---|---|---|---|---|
| **Posture** | Normal | 0 | | | | | | | |
| | Decreasing activity, Decreasing normal behaviour, pilo-erection | 3 | | | | | | | |
| | Huddled , not moving in cage | 5 | | | | | | | |
| **Temperature Chnge** | Increase in body temperature above 2°C (n=38.9) | 5 | | | | | | | |
| **Weight Change** | Decrease in body weight of more than 10% | 10 | | | | | | | |
| **Respiration** | Normal | 0 | | | | | | | |
| | Increased or Decreased | 2 | | | | | | | |
| | Laboured, breathing through mouth | 10 | | | | | | | |
| | Cough or sneeze | 2 | | | | | | | |
| **Feces + Urine** | Normal consistency volume / Soft normal stool | 0 | | | | | | | |
| | Feces absent or dry / Decreased urine output / Cloudy urine | 2 | | | | | | | |
| | Wet pasty / Small very dry stool / dark stool | 2 | | | | | | | |
| | Liquid stool / blood in stool or urine<br>No urine > twice | 10 | | | | | | | |
| **Food + water** | Normal eating / drinking | 0 | | | | | | | |
| | Mildly decreased E /D 25% | 1 | | | | | | | |
| | Moderately decreased E / D 50 % | 3 | | | | | | | |
| | Severely decreased E/D 75% | 4 | | | | | | | |
| | Seriously decreased - refusing all food, dehydration apparent > 2 days | 10 | | | | | | | |
| **Recumbent** | No symptoms | 0 | | | | | | | |
| | Huddled on camera, active when cage opened | 3 | | | | | | | |
| | Lies down but moves around | 15 | | | | | | | |
| | Lies down and won't move | 25 | | | | | | | |
| **Attitude** | Normal | 0 | | | | | | | |
| | Mildly depressed, responds to treats and toys | 1 | | | | | | | |
| | Moderately depressed, response requires prodding, loses interest in treats and toys, | 3 | | | | | | | |
| | Severely depressed, no interest in treats, does not respond to human presence | 10 | | | | | | | |
| **Other** | Flushed appearance to skin | 2 | | | | | | | |
| | Nasal discharge | 2 | | | | | | | |
| | Visible Rash | 5 | | | | | | | |
| | Cyanosis | 5 | | | | | | | |
| | Haemorrhage — Subcutaneous | 10 | | | | | | | |
| | Haemorrhage — Orifices | 15 | | | | | | | |
| **Total score*** | | 0-64* | | | | | | | |
| **BODY Weight** | | | | | | | | | |
| **SCAN Temp** | | | | | | | | | |
| **Rectal Temp** | | | | | | | | | |

**** The PI or co-investigator will consult with a veterinarian to make a decision regarding euthanasia when a total score of 25 is reached***

**** The control animals will only be allowed to reach a score of 20 before a decision is made regarding euthanasia.***
